# Causal Inference of Different Smoke Exposure Statuses and Influenza Risk: Insights From a Mendelian Randomization Study

**DOI:** 10.1111/crj.70083

**Published:** 2025-05-13

**Authors:** Yanqi Guo, Haixia Chen, Shijie Wu, Jiesen Zhou, Zhihua Chen

**Affiliations:** ^1^ Key Laboratory of Respiratory Disease of Zhejiang Province, Department of Respiratory and Critical Care Medicine Second Affiliated Hospital of Zhejiang University School of Medicine Zhejiang Hangzhou China; ^2^ Center for Nephrology and Clinical Metabolomics and Division of Nephrology, Shanghai Tenth People's Hospital Tongji University School of Medicine Shanghai China

**Keywords:** influenza, Mendelian randomization analysis, pneumonia, tobacco smoke pollution

## Abstract

**Introduction:**

Previous observational studies have suggested a potential association between smoking exposure and influenza infection risk. However, the impact of different smoke exposure statuses on susceptibility to influenza infection remains insufficiently explored. This study employs Mendelian randomization analysis to investigate the causal relationship between smoking exposure statuses, including current tobacco use, household smoking exposure, past smoking history, and the risk of influenza infection.

**Methods:**

The summary‐level data for this study were obtained from the FinnGen Consortium R11 and Neale Lab, both outcomes and exposures. To ensure robust results, we employed multiplicative random‐effects inverse variance weighting, MR‐Egger, and weighted median (WM) methods to analyze single‐nucleotide polymorphisms (SNPs). We also conducted Cochran's *Q* test, MR‐PRESSO, and the MR‐Egger intercept test to assess heterogeneity and horizontal pleiotropy, ensuring accurate and reliable findings.

**Results:**

Our analysis demonstrated that elevated exposure to current tobacco smoking causally increased the risk of influenza infection, with (OR = 2.032, 95% CI 1.672–2.538, *p* < 0.001) or without pneumonia (OR = 2.081, 95% CI 1.824–2.338, *p* = 0.015). No reverse causal relationship was found, and no bidirectional effects were observed for past smoking (OR = 1.108, 95% CI 0.543–2.258, *p* = 0.779) or household exposure (OR = 1.127, 95% CI −0.209–2.462, *p* = 0.939).

**Conclusion:**

This analysis identified a significant causal association between current tobacco smoking and increased risk of influenza infection. However, no significant association was observed for other smoking exposures (e.g., former or household smoking). These findings emphasized the importance of considering different types of smoking exposure in clinical influenza prevention and treatment strategies.

## Graphical abstract

This study employs Mendelian randomization to explore the causal relationship between smoking exposure and influenza risk. Current smoking significantly increases the risk of influenza, with or without pneumonia. No reverse causality was found, and past smoking or household exposure showed no bidirectional effects.

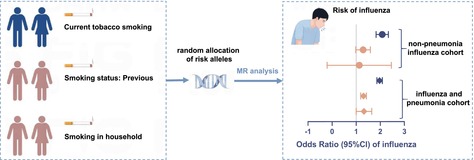

## 1 Introduction

Influenza is an acute viral infection that primarily targets the respiratory system and spreads rapidly between individuals [1]. The World Health Organization reports that seasonal influenza affects nearly one billion people worldwide each year, with 3 to 5 million cases classified as severe. This illness leads to an estimated 290 000 to 650 000 respiratory‐related deaths annually [2]. Children under five experience the highest rates of influenza‐related hospital admissions, while most influenza‐attributable deaths in hospitals occur among individuals aged 65 and older with co‐morbidities [3]. Previous research has shown an association between influenza and pneumonia. Influenza virus infection can disrupt both innate and adaptive immune responses, making the host more susceptible to secondary bacterial infections and elevating the likelihood of developing bacterial pneumonia as a secondary complication [4]. Additionally, patients hospitalized with influenza‐related pneumonia experience higher mortality rates, which significantly contributes to the worldwide burden of pneumonia.

Smoking remains the most significant preventable behavioral risk to health. Long‐term exposure to tobacco smoke and household air pollution contributes to numerous chronic respiratory diseases. In 2018, over one in five adults in European Union countries continued to smoke daily. The TackSHS project emphasized the dangers of passive smoking in Europe, revealing that second‐hand smoke affects 31% of the population. A considerable portion of those exposed to smoking within the household have increased health risks [5].

The potential bidirectional causal association between influenza infection and smoking exposure has been the subject of extensive investigation over several decades. However, randomized clinical trials have yielded inconsistent evidence. For instance, a study by Cruijff et al. found no significant correlation between current tobacco smoking and either serological or clinical influenza [6]. In contrast, two studies conducted in 2014 identified tobacco smoking status as a risk factor for influenza infection [7, 8]. As for the susceptibility to influenza infection in individuals with previous smoking exposure and those exposed to household smoking, there is a lack of high‐quality observational studies and randomized clinical trials. Consequently, whether a bidirectional causal relationship exists between various smoking exposure statuses and the risk of influenza remains ambiguous.

As a well‐established epidemiological method, Mendelian randomization (MR) analysis is designed to reduce unmeasured confounding in observational studies and to overcome the limitations of specificity in randomized trials [9]. In this study, we utilized genome‐wide association study (GWAS) data to clarify the bidirectional causal effects of different smoking exposure statuses on influenza infection and to explore potential disparities in smoking environments, specifically focusing on current tobacco users, former smokers, and individuals exposed to household smoking.

## 2 Materials and Methods

### 2.1 Study Design

Our study estimated the bidirectional causal effects between various smoking exposure statuses and the risk of influenza and influenza pneumonia infection, following the STROBE‐MR guidelines [10]. We categorized influenza into mild cases and more severe cases of influenza pneumonia based on severity. We subsequently employed these features to evaluate the causal relationship between susceptibility to influenza and influenza pneumonia and different smoking statuses, including current tobacco smokers, former smokers, and individuals living in households with smokers.

### 2.2 Pooled Summary Statistic of GWAS

To identify potential instrumental variables for influenza and smoking exposure statuses, we employed summary statistics from genome‐wide association studies undertaken by the FinnGen Consortium and Neale Lab. The GWAS for influenza (not pneumonia) as a regular influenza group with 6092 cases and 378 292 controls, while influenza pneumonia cohort, representing the severe influenza group, comprised 75 441 cases and 378 292 controls from the FinnGen Consortium R11 [11, 12]. GWAS summary statistics for smoking exposure statuses were obtained from Neale Lab, which included 337 030 participants who were current tobacco smokers, 336 024 participants who were former smokers, and 311 142 participants from households with smoking.

The GWAS data utilized in this Mendelian randomization investigation were sourced from https://gwas.mrcieu.ac.uk/ and https://www.finngen.fi/en/access_results.

### 2.3 Instrument Variables Selection Criteria

MR analyses depend on the GWAS of exposures and outcomes to enable the analysis of bidirectional causal effects from phenotype studies to genotype studies [13]. Mendelian randomization model requires to select potential instrumental variable, which have three core assumptions. Firstly, the SNP indicates a robust connection with the exposure factor. Secondly, MR relies on the independence assumption, meaning the SNP should be independent of confounders. Thirdly, the SNPs affect the outcome solely through the exposure, without horizontal pleiotropy [10, 14, 15]. To select suitable SNPs as instrumental variables (IVs), several guidelines are followed. (1) Identification of potential SNPs: Index SNPs correlated with the exposure were chosen using a significance threshold of *p* < 5 × 10^−6^, indicating a stronger likelihood of relevance to the exposure of interest [16]. (2) Perform linkage disequilibrium (LD) clamping: LD indicates the nonrandom correlation of alleles located at distinct loci. Evaluation is conducted using two parameters: *r* ^2^ and kb, with criteria established at *r* ^2^ < 0.001 and a window size of 10 000 kb. (3) Based on the summary statistics from GWAS conducted using the FinnGen database and Neale Lab, we evaluated the relationships between index SNPs and possible confounders for smoking exposure traits, including eosinophil count, C‐reactive protein levels, platelet count, monocyte count, lymphocyte count, and neutrophil count. For the influenza trait, the potential confounders included MHPG levels, neutrophil count, forced expiratory volume, and smoking initiation. To mitigate potential covariance issues, we excluded index SNPs associated with these confounders (*p* < 1E−5). Detailed information regarding the confounders and the removed SNPs is provided in Table S2. (4) Calculation of F‐statistics: To mitigate weak instrument bias, we required that all instruments have an F‐statistic greater than 10. F‐statistics is calculated to assess the strength of the association between the remaining SNPs and the exposure. The F‐statistic for each SNP was computed using the formula: *F = β (exposure)* ^*2*^ */SE (exposure)* ^*2*^, where *β (exposure)* represents the estimated effect size of the SNP on the exposure, and *SE (exposure)* is its standard error. This approach ensures that only SNPs with sufficiently strong associations are retained as valid instruments [17]. (5) Exclusion of palindrome SNPs: The harmonies function from the TwoSampleMR R package was used to align effect alleles between the exposure and outcome datasets. Palindrome SNPs were excluded by inferring the alleles based on allele frequency data [18].

### 2.4 Statistical Analysis

The bidirectional MR analysis conducted across the FinnGen and Neale Lab cohorts utilized four different MR methods, each with distinct assumptions: inverse variance weighting (IVW), weighted median, MR‐Egger, and weighted mode. The main approach employed for this analysis was inverse variance weighting, recognized as the most dependable method when heterogeneity or pleiotropy is not present [19]. The MR‐Egger regression intercept was applied to evaluate horizontal pleiotropy in the Mendelian randomization analyses, with any deviation from zero indicating potential directional pleiotropy [20]. MR‐PRESSO test systematically identified and excluded SNPs with horizontal pleiotropy across all Mendelian randomization analyses through its outlier correction method. To evaluate result robustness, leave‐one‐out (LOO) sensitivity analyses determined whether any individual SNP disproportionately influenced causal estimates. Following the removal of pleiotropic SNPs by MR‐PRESSO and exclusion of influential outliers (including effect direction‐reversing variants) in LOO analysis, Cochran’s *Q* test assessed residual heterogeneity in the final instrument set, with a *p* > 0.05 indicating no statistically significant heterogeneity across the included studies. All statistical analyses were performed using R version 4.1.1 (R Foundation for Statistical Computing, Vienna, Austria). The Mendelian randomization analyses were conducted with several R packages, including TwoSampleMR (version 0.6.7), ieugwasr (version 0.1.5), MendelianRandomization (version 0.10.0), vroom (version 1.6.5), tidyr (version 1.3.1), dplyr (version 1.1.4), and data.table (version 1.16.0).

## 3 Results

### 3.1 Overview of Demographic Attributes

**FIGURE 1:**
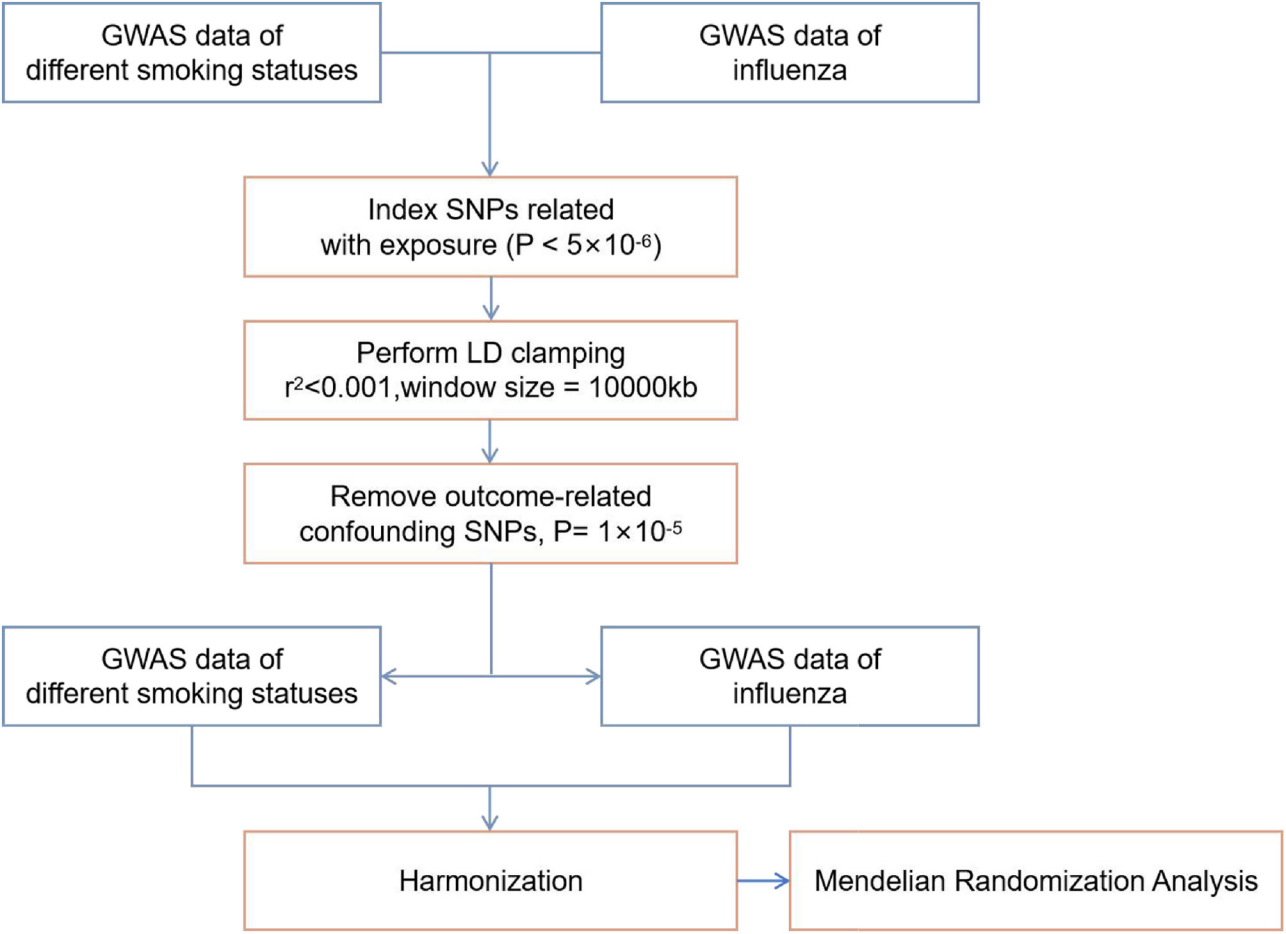
The flowchart for selecting instrumental variables from GWAS data. Abbreviations: SNP, single nucleotide polymorphism; OR, odds ratio; CI, confidence interval.

To accurately assess genetic difference in smoking exposure and influenza, the process of instrument viables selection is presented in Figure 1. The comprehensive assessment of influenza risk was based on two cohorts: the non‐pneumonia influenza cohort and the influenza pneumonia cohort, the latter being considered a more severe form. The overview of fundamental properties of the GWAS data from these cohorts is available in Table 1.

**TABLE 1:** The summary information on analyzed traits and GWAS consortia.

Trait	Consortium	Ethnicity	Sample size	Web‐link
Current tobacco smoking	Neale Lab	European	337 030	https://www.nealelab.is/uk‐biobank
Smoking status: previous	Neale Lab	European	336 024	https://www.nealelab.is/uk‐biobank
Smoking in household	Neale Lab	European	311 142	https://www.nealelab.is/uk‐biobank
Influenza (not pneumonia)	FinnGen	European	384 384	https://www.finngen.fi/fi
Influenza and pneumonia	FinnGen	European	453 733	https://www.finngen.fi/fi

Abbreviation: SNP, single nucleotide polymorphism.

### 3.2 Selecting of Instrumental Variables for Smoking Exposure Statuses

In the current tobacco smoking cohort, 3635 SNPs were correlated with smoking exposure in the GWAS conducted by the Neale Lab. Based on the IV selection criteria, 95 independent SNPs through LD pruning. After confounder adjustment, effect allele harmonization, and MR‐PRESSO pleiotropy testing, 77 SNPs qualified as IVs for influenza (excluding pneumonia) as the outcome, while 70 SNPs were retained for influenza and pneumonia as the outcome. In the previous smoking history cohort, 4045 SNPs were associated with smoking exposure. After the procedure of selecting IVs and MR‐PRESSO test, 72 SNPs were deemed eligible IVs for influenza (excluding pneumonia) as the outcome, while 71 SNPs were retained for influenza and pneumonia as the outcome. For the household smoking exposure cohort, 238 SNPs were associated with smoking exposure. Ten SNPs passed the entire IV selection process when both influenza risk cohorts were used as outcomes. The F‐statistics for all selected genetic IVs of smoking exposure status exceeded the weak instrument threshold (F‐statistic < 10), indicating that these IVs are strong genetic variants suitable for MR study (Table S4). Table S1 shows the number of SNPs preserved following each step of IVs selection. Table S3 presents the characteristics of SNPs used in MR analyses following MR‐PRESSO testing.

### 3.3 Assessments of Causal Effect of Smoking Exposure Statuses on Influenza Risk

**FIGURE 2:**
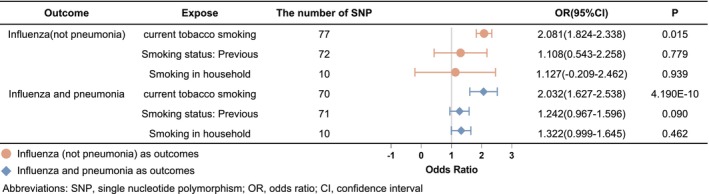
Forest plot of the causal effect of genetically predicted smoking exposure statuses on influenza risk using the IVW method. Abbreviations: SNP, single nucleotide polymorphism; OR, odds ratio; CI, confidence interval.

The causal relationship between smoking exposure and the risk of influenza infection was evaluated using Mendelian randomization. The IVW MR analysis revealed considerable variations in the risk of influenza infection across different smoking exposure settings. Notably, the odds ratio (OR) of influenza (non‐pneumonia) for smoking exposure was 2.081 (95% CI 1.824–2.338, *p* = 0.015) in the current tobacco smoking cohort, 1.1.08 (95% CI 0.543–2.258, *p* = 0.779) in the previous smoking history cohort, and 1.127 (95% CI −0.209–2.462, *p* = 0.939) in the household smoking exposure cohort. Furthermore, a similar trend was observed with a more severe form of influenza, where the OR for influenza and pneumonia due to smoking exposure was 2.032 (95% CI 1.627–2.538, *p* = 4.190E−10) in the current tobacco smoking cohort, 1.242 (95% CI 0.967–1.596, *p* = 0.090) in the previous smoking history cohort, and 1.322 (95% CI 0.009–1.645, *p* = 0.462) in the household smoking exposure cohort. MR analysis provided strong evidence that the current tobacco smoking cohort correlates with an elevated risk of influenza. The forest plots in Figure 2 illustrate the assessments of the causal effect of smoking exposure on influenza risk, using the IVW analysis.

Table 2 illustrates the sensitivity analyses, showing that the exposure in the current tobacco smoking group has a completely consistent direction of causal relationship. However, the direction of the odds ratios in MR‐Egger was inconsistent for the exposure in the household smoking group, with values of 0.032 and 0.486 for outcomes with and without pneumonia, respectively. Cochran's *Q* test demonstrated no significant heterogeneity (Table 2). Additionally, both the MR‐Egger intercept tests (*p*_ple_ > 0.05 in all groups; Table 2) and leave‐one‐out sensitivity analyses (Figures S1a–S6a) consistently showed no evidence of horizontal or directional pleiotropy, and no single SNP was identified as the sole driver of the observed associations. The forest plot, regression lines, and funnel plot for the Mendelian randomization analyses of smoking exposure statuses on influenza risk are illustrated in Figures S1 to Figures S6.

**TABLE 2:** Sensitivity analysis of different smoking exposure statuses on the risk of influenza in TS‐MR analyses.

Outcome	Influenza (not pneumonia)									Influenza and pneumonia								
Exposure	Current tobacco smoking			Smoking status: Previous			Smoking in household			Current tobacco smoking			Smoking status: Previous			Smoking in household		
	OR	95%CI	*p*	OR	95%CI	*p*	OR	95%CI	*p*	OR	95%CI	*p*	OR	95%CI	*p*	OR	95%CI	*p*
IVW method	2.081	1.824–2.338	0.015	1.108	0.543–2.258	0.779	1.127	−0.209‐2.462	0.939	2.032	1.627–2.538	4.190E‐10	1.242	0.967–1.596	0.090	1.322	0.999–1.645	0.462
Weighted median	1.828	1.460–2.196	0.163	1.056	0.378–2.949	0.918	0.234	−1.213‐1.682	0.394	1.978	1.428–2.739	5.613E‐04	1.264	0.897–1.779	0.18	0.711	0.267–1.155	0.513
MR‐Egger	1.075	0.300–1.851	0.936	0.689	0.038–12.472	0.802	0.032	−2.342‐2.407	0.219	1.256	0.612–2.574	0.536	1.251	0.438–3.576	0.677	0.486	−0.132‐1.105	0.321
Weighted mode	1.154	0.356–1.953	0.879	1.064	0.095–11.979	0.960	0.821	−1.469‐1.751	0.300	1.699	0.854–3.377	0.135	1.405	0.543–3.630	0.485	0.765	0.242–1.287	0.662
Heterogeneity	Cochrane's *Q* = 68.829; P_het_ = 0.708			Cochrane's *Q* = 71.183; P_het_ = 0.471			Cochrane's *Q* = 15.445; P_het_ = 0.079			Cochrane's *Q* = 84.165; P_het_ = 0.103			Cochrane's *Q* = 86.145; P_het_ = 0.092			Cochrane's *Q* = 4.854; P_het_ = 0.846		
Pleiotropy	Egger_Intercept = 5.939E‐03; P_ple_ = 0.443			Egger_Intercept = 3.449E‐03; P_ple_ = 0.741			Egger_Intercept = 0.030; P_ple_ = 0.135			Egger_Intercept = 4.176E‐03; P_ple_ = 0.172			Egger_Intercept = −5.234E‐05; P_ple_ = 0.989			Egger_Intercept = 8.414E‐03; P_ple_ = 0.115		

*Note:* MR‐Egger was employed to assess pleiotropy. Neither pleiotropy nor heterogeneity was detected across all analyses.

Abbreviations: CI, confidence interval; IVW, inverse variance weighted method; OR, odds ratio.

### 3.4 Selecting of Instrumental Variables for Influenza Risk

Similar selection procedures were used to select the genetic IVs for influenza risk. In the non‐pneumonia influenza cohort, 84 SNPs were correlated with influenza in the FinnGen GWAS. Following a series of instrumental variable selection steps and application of MR‐PRESSO, 10 SNPs were ultimately selected as IVs for the analyses of two distinct smoking exposure outcomes: previous smoking history and household smoking exposure. For the outcome of current tobacco smoking, eight SNPs were retained as IVs. In the influenza pneumonia cohort, 1776 SNPs were correlated with influenza. When both current tobacco smoking and household smoking exposure were used as two distinct smoking exposure outcomes, 28 SNPs passed the entire IVs selection process. For the outcome of previous smoking history, 29 SNPs were deemed eligible as IVs. The F‐statistics for all selected genetic IVs of influenza risk were above the weak instrument threshold (F‐statistic < 10), indicating that these IVs are strong genetic variants suitable for MR study (Table S4). Table S1 shows the number of SNPs preserved following each step of IVs selection. Table S3 presents the characteristics of SNPs used in MR analyses after MR‐PRESSO testing.

### 3.5 Assessments of Causal Effect of Influenza Risk on Smoking Exposure Statuses

**FIGURE 3:**
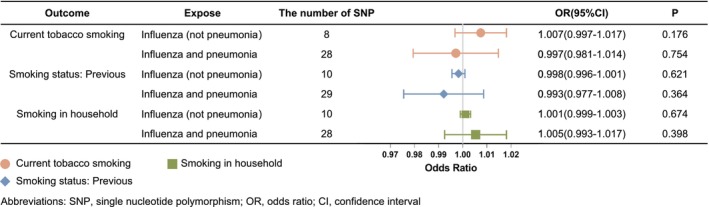
Forest plot of the causal effect of genetically predicted influenza risk on smoking exposure statuses using the IVW method.

Figure 3 illustrates the causal effect of influenza infection on various smoking exposure conditions, as determined by Mendelian randomization. The IVW MR analysis did not reveal significant disparities in the impact of influenza infection across different smoking exposure statuses.

The OR of current smokers for influenza infection was 1.007 (95% CI 0.997–1.017, *p* = 0.176) in influenza (not pneumonia) cohort and 0.997 (95% CI 0.981–1.014, *p* = 0.754) in the influenza and pneumonia cohort. For former smokers, the OR was 0.998 (95% CI 0.996–1.001, *p* = 0.621) in the influenza (not pneumonia) cohort and 0.993 (95% CI 0.977–1.008, *p* = 0.364) in the influenza and pneumonia cohort. Similarly, for household smoking exposure, the OR was 1.001 (95% CI 0.999–1.003, *p* = 0.674) in the first cohort and 1.005 (95% CI 0.093–1.017, *p* = 0.398) in the second cohort. The forest plots in Figure 3 illustrate the assessments of the causal effect of influenza infections on smoking exposure conditions, using the IVW analysis.

Table 3 demonstrates the sensitivity analyses, showing that the exposure in influenza with or without pneumonia has a most consistent direction of causal effect. Cochran’s *Q* test demonstrated no significant heterogeneity (Table 3) Both the MR‐Egger intercept tests (*p*_ple_ > 0.05 in all group; Table 3) and leave‐one‐out sensitivity analyses (Figures S7a–S12a) consistently showed no evidence of horizontal or directional pleiotropy, and no single SNP was identified as the sole driver of the observed associations. The forest plot, regression lines, and funnel plot for the Mendelian randomization analyses of influenza risk on various smoking exposure statuses are displayed in Figures S7 through Figures S12.

**TABLE 3:** Sensitivity analysis of influenza on the smoking exposure statuses in TS‐MR analysis.

Outcome	Current tobacco smoking						Smoking status: previous						Smoking in household					
Exposure	Influenza (not pneumonia)			Influenza and pneumonia			Influenza (not pneumonia)			Influenza and pneumonia			Influenza (not pneumonia)			Influenza and pneumonia		
	OR	95%CI	*p*	OR	95%CI	*p*	OR	95%CI	*p*	OR	95%CI	*p*	OR	95%CI	*p*	OR	95%CI	*p*
IVW method	1.007	0.997–1.017	0.176	0.997	0.981–1.014	0.754	0.998	0.996–1.001	0.621	0.993	0.977–1.008	0.364	1.001	0.999–1.003	0.674	1.005	0.993–1.017	0.398
Weighted median	1.007	0.997–1.018	0.173	0.991	0.967–1.015	0.441	0.999	0.995–1.002	0.752	0.991	0.971–1.011	0.382	0.998	0.995–1.001	0.505	1.007	0.991–1.023	0.401
MR‐Egger	1.003	0.988–1.018	0.747	1.016	0.983–1.050	0.356	0.998	0.994–1.003	0.735	0.992	0.961–1.024	0.629	0.997	0.993–1.000	0.378	1.004	0.978–1.030	0.776
Weighted mode	1.007	0.998–1.017	0.183	0.983	0.938–1.031	0.489	0.998	0.994–1.003	0.751	0.988	0.961–1.016	0.404	0.998	0.995–1.000	0.468	1.011	0.990–1.031	0.329
Heterogeneity	Cochrane's *Q* = 11.908; P_het_ = 0.104			Cochrane's *Q* = 30.363; P_het_ = 0.298			Cochrane's *Q* = 8.385; P_het_ = 0.496			Cochrane's *Q* = 35.827; P_het_ = 0.147			Cochrane's *Q* = 7.553; P_het_ = 0.580			Cochrane's *Q* = 32.633; P_het_ = 0.044		
Pleiotropy	Egger_Intercept = 1.547E‐03; P_ple_ = 0.464			Egger_Intercept = −9.717E‐04; P_ple_ = 0.214			Egger_Intercept = 3.96E‐05; P_ple_ = 0.971			Egger_Intercept = 4.065E‐05; P_ple_ = 0.956			Egger_Intercept = 1.264E‐03; P_ple_ = 0.141			Egger_Intercept = 5.659E‐04; P_ple_ = 0.907		

*Note:* MR‐Egger intercept was employed to assess pleiotropy. Neither pleiotropy nor heterogeneity was detected across all analyses.

Abbreviations: CI, confidence interval; IVW, inverse variance weighted method; OR, odds ratio.

## 4 Discussion

Influenza is a widespread respiratory virus that rapidly spreads during seasonal epidemics, causing significant global economic burden [2]. Up to 40% of community‐acquired pneumonia (CAP) cases are linked to viral infections. Vaughn et al. recommend mandatory influenza testing for all pneumonia patients during times of high community viral prevalence [21]. Previous research has shown that smoking exposure increases the risk of respiratory infections, leading to more severe outcomes [22]. Cigarette smoking also as a risk factor increase the susceptibility to influenza [23]. Several potential mechanisms may underlie the increased susceptibility to influenza infection due to cigarette smoking exposure, including structural alterations in the respiratory tract and a compromised immunologic defense system [24]. Experimental data from animal models suggest that cigarette smoke predominantly impacts antiviral immune‐inflammatory responses by elevating the expression of key markers, including interleukin‐6 and tumor necrosis factor‐alpha. Despite this, secondary immune protection, such as the formation of influenza‐specific memory responses, remains unaffected [25]. The current study reveals a novel mechanism by which chemical compounds in cigarette smoke trigger driven thrombo‐inflammatory response, involving the activation of protease‐activated receptors and mediated by platelet–neutrophil cross‐communication, which induces the formation of neutrophil–platelet aggregates (NPAs) and the release of neutrophil extracellular traps (NETs) during influenza pneumonia. However, a case note‐based investigation of pandemic H1N1 infection has shown conflicting results, indicating that smoking was not identified as a risk factor. Moreover, a randomized clinical trial has also indicated that smokers did not exhibit a higher incidence of serological or clinical influenza compared to non‐smokers [9]. Mendelian randomization can provide deeper genetic insights into the causal association between smoking exposure and influenza infection by analyzing this relationship in both directions.

Current research remains unclear on whether individuals with different smoking statuses experience similar increases in susceptibility to influenza and pneumonia. A retrospective observational cohort study assessed the odds ratios for influenza‐related medical encounters (IRME) among different smoking status groups. Former smokers and never smokers had lower odds of influenza‐related hospitalization compared to current smokers [26]. Meanwhile, a study of 15 354 children with severe respiratory infections, those diagnosed with H3N2 influenza were more likely to have a household smoker (26%, 54/208) compared to children from non‐smoking parents (16%, 158/960). Our Mendelian randomization analysis explored the potential causal effect of influenza infection on different types of smoking exposure, including current tobacco use, former smoking, and household smoke exposure. The results revealed a potential causal relationship between influenza infection and increased prevalence of current tobacco smoking. In contrast, no significant association was observed for former smokers or individuals exposed to household smoking. These findings align with prior meta‐analyses, which have also indicated that current smokers are at an elevated risk of contracting influenza, encompassing influenza‐like illness and microbiologically proven influenza [27]. However, inconsistencies remain regarding the impact of household smokers. The US Surgeon General's report highlights passive smoking as a significant contributor to lower respiratory infections among youngsters [28]. Additionally, a meta‐analysis conducted by Shi et al. identified maternal smoking as a significant risk factor for RSV‐associated lower respiratory tract infections in children (OR 1.36; 95% CI 1.24–1.50), while another meta‐analysis by Laura L. Jones et al. demonstrated that smoking by any household member increases the risk of lower respiratory infections in infants, with the strongest association observed in cases of bronchiolitis [29, 30]. The inconsistent causal effects of household smoking exposure on the risk of influenza infection indicate that age‐stratified endpoints in trials may introduce bias. To enhance the reliability of these findings, further validation through additional clinical trials or meta‐analyses targeting various age group endpoints is essential. These studies highlight the importance of recognizing disparities in influenza susceptibility across different smoking exposure statuses, which is essential for refining healthcare strategies tailored to individual patient needs. Furthermore, researchers must consider these variables in future studies to enhance the accuracy of their findings and improve the development of preventive measures and treatments.

Our study has several strengths. First, it is the first to utilize Mendelian randomization analyses to elucidate the bidirectional causal relationship between susceptibility to influenza and influenza pneumonia and various smoking exposure statuses. Additionally, we revealed potential differences in the smoking environment that may influence this causal effect. Third, we utilized summary statistics from GWAS on current tobacco smokers, former smokers, and individuals exposed to household smoking. This genetic approach helps validate the findings and mitigate observed disparities by accounting for confounding factors. To ensure the robustness of our Mendelian randomization analyses, we employed several methods to minimize the violation of key assumptions, including LD clumping, the exclusion of outcome‐related confounding SNPs, and selecting IVW as primary methods to present results free from heterogeneity and pleiotropy. Finally, our research our research provides valuable insights into the link between smoking exposure and influenza, contributing to the development of preventive and therapeutic strategies for influenza.

Our research still has several limitations. First, influenza and influenza pneumonia were assessed only in terms of disease severity, and due to data constraints, we were unable to perform Mendelian randomization analyses to investigate potential causal differences based on factors such as age and sex. Second, all GWAS data were derived from individuals of European ancestry, and the applicability of the results to broader populations requires further validation. Furthermore, when selecting index SNPs associated with exposure, we opted for a threshold of *p* < 5 × 10^−6^, rather than the conventional *p* < 5 × 10^−8^, to balance statistical rigor with practical considerations. Therefore, further clinical trials are required to validate the relationship between smoking exposure and susceptibility to influenza.

## 5 Conclusion

This study provides evidence that current tobacco smoking, but not former smoking or household smoke exposure, is causally associated with increased susceptibility to influenza. Confirmation of these findings through large‐scale prospective investigations is warranted.

## Author Contributions

Study design and conceptualization: YG. Statistical analysis and interpretation: YG, HC, SW. Manuscript writing: YG with revisions from HC, SW, and ZC. Supervision: ZC and JZ.

## Ethics Statement

Not applicable.

## Consent

Not applicable.

## Conflicts of Interest

The authors declare no conflicts of interest.

## Supporting information


**Table S1** Number of index SNPs retained at each step of instrumental variable selection.

**Table S2** Index SNPs significant associated with the potential confounders.

**Table S3** Results of the MR‐PRESSO testing.

**Table S4** SNP summary for MR analysis (including F‐statistics and FDR‐adjusted *p* values)

**Figure S1** Mendelian randomization analysis of current tobacco smoking on the risk of influenza (excluding pneumonia).

**Figure S2** Mendelian randomization analysis of current tobacco smoking on the risk of influenza and pneumonia.

**Figure S3** Mendelian randomization analysis of previous smoking history on the risk of influenza (excluding pneumonia).

**Figure S4** Mendelian randomization analysis of previous smoking history on the risk of influenza and pneumonia.

**Figure S5** Mendelian randomization analysis of household smoking exposure on the risk of influenza (excluding pneumonia).

**Figure S6** Mendelian randomization analysis of household smoking exposure on the risk of influenza and pneumonia.

**Figure S7** Mendelian randomization analysis of influenza (not pneumonia) infection on current tobacco use cohort.

**Figure S8** Mendelian randomization analysis of influenza (not pneumonia) infection on previous smoking cohort.

**Figure S9** Mendelian randomization analysis of influenza (not pneumonia) infection on household smoking exposure cohort.

**Figure S10** Mendelian randomization analysis of influenza and pneumonia infection on current tobacco use cohort.

**Figure S11** Mendelian randomization analysis of influenza and pneumonia infection on previous smoking cohort.

**Figure S12** Mendelian randomization analysis of influenza and pneumonia infection on household smoking exposure cohort.

## Data Availability

The data that support the findings of this study are available from the corresponding author upon reasonable request. The majority of results are contained in the supplementary information. The accession websites for the datasets are enumerated in the Table 1. Supplementary results, encompassing the data utilized for all figures and tables, can be obtained upon request from the lead contact. This publication does not present original code.
