# Supplementary material for: Causal Inference of Different Smoke Exposure Statuses and Influenza Risk: Insights From a Mendelian Randomization Study
Source: Clin Respir J. 2025 May 13;19(5):e70083. doi: 10.1111/crj.70083 (PMC12075745; doi:10.1111/crj.70083)
Supplement: Supplementary file 6 — Figure S2 Mendelian randomization analysis of current tobacco smoking on the risk of influenza and pneumonia. [file CRJ-19-e70083-s003.pdf]

Figure S2. Mendelian randomization analysis of current tobacco smoking on the risk of influenza and pneumonia.

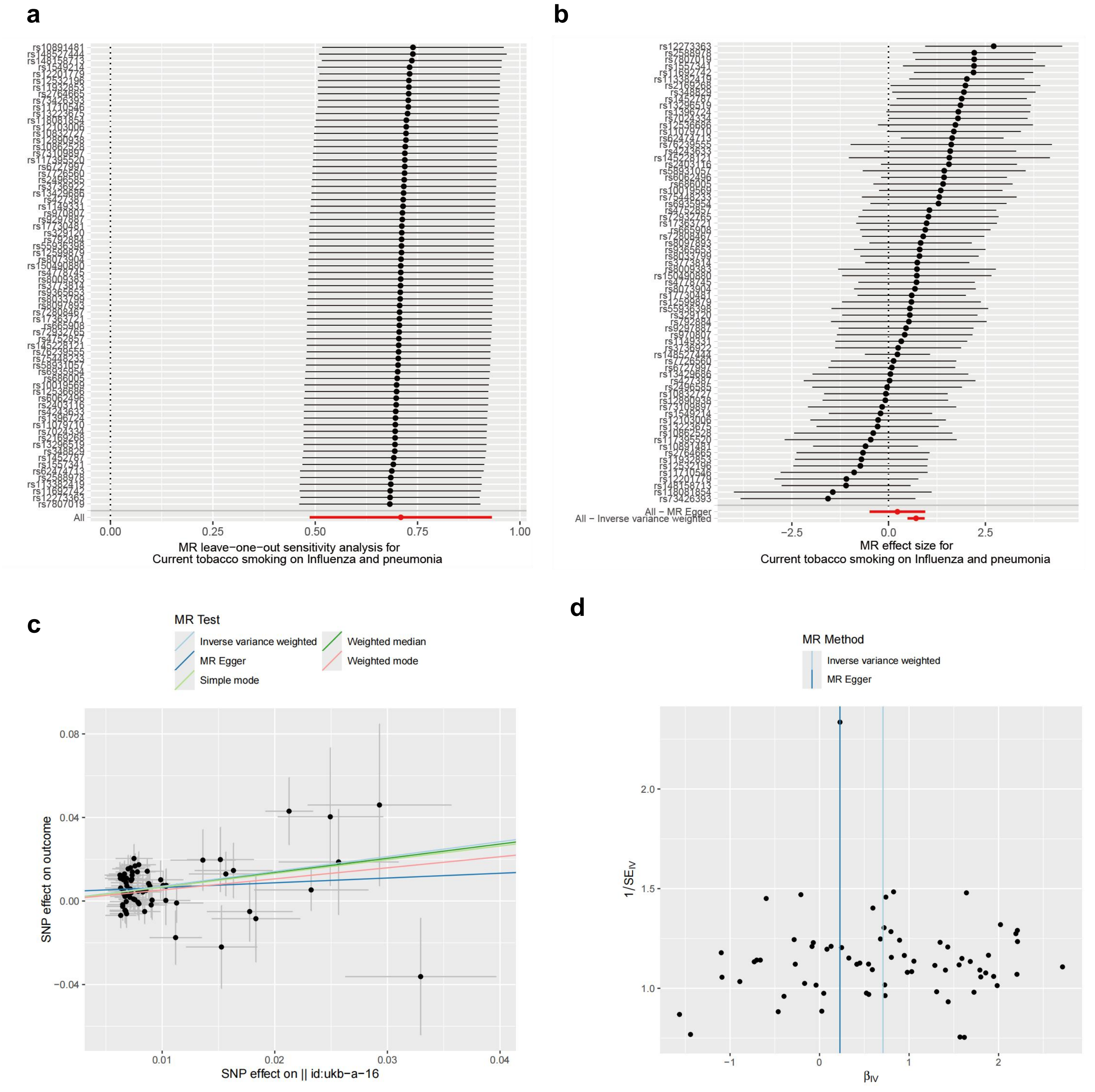

Figure S2.Mendelian randomization analysis of current tobacco smoking on the risk of influenza and pneumonia. (a) Leave-one-out analysis of MR test from current tobacco smoking on the risk of influenza and pneumonia. **(b)** Forest plot showing the effect estimates of individual SNPs associated with current tobacco smoking on the risk of influenza and pneumonia. **(c)** Regression lines representing Mendelian Randomization (MR) test results for the causal effect of current smoking on influenza and pneumonia risk. **(d)** Funnel plot illustrating the distribution of individual SNP estimates for current smoking on influenza and pneumonia risk, used to assess potential bias or heterogeneity.
