# Supplementary material for: Causal Inference of Different Smoke Exposure Statuses and Influenza Risk: Insights From a Mendelian Randomization Study
Source: Clin Respir J. 2025 May 13;19(5):e70083. doi: 10.1111/crj.70083 (PMC12075745; doi:10.1111/crj.70083)
Supplement: Supplementary file 7 — Figure S3 Mendelian randomization analysis of previous smoking history on the risk of influenza (excluding pneumonia). [file CRJ-19-e70083-s002.pdf]

Figure S3. Mendelian randomization analysis of previous smoking history on the risk of influenza (excluding pneumonia).

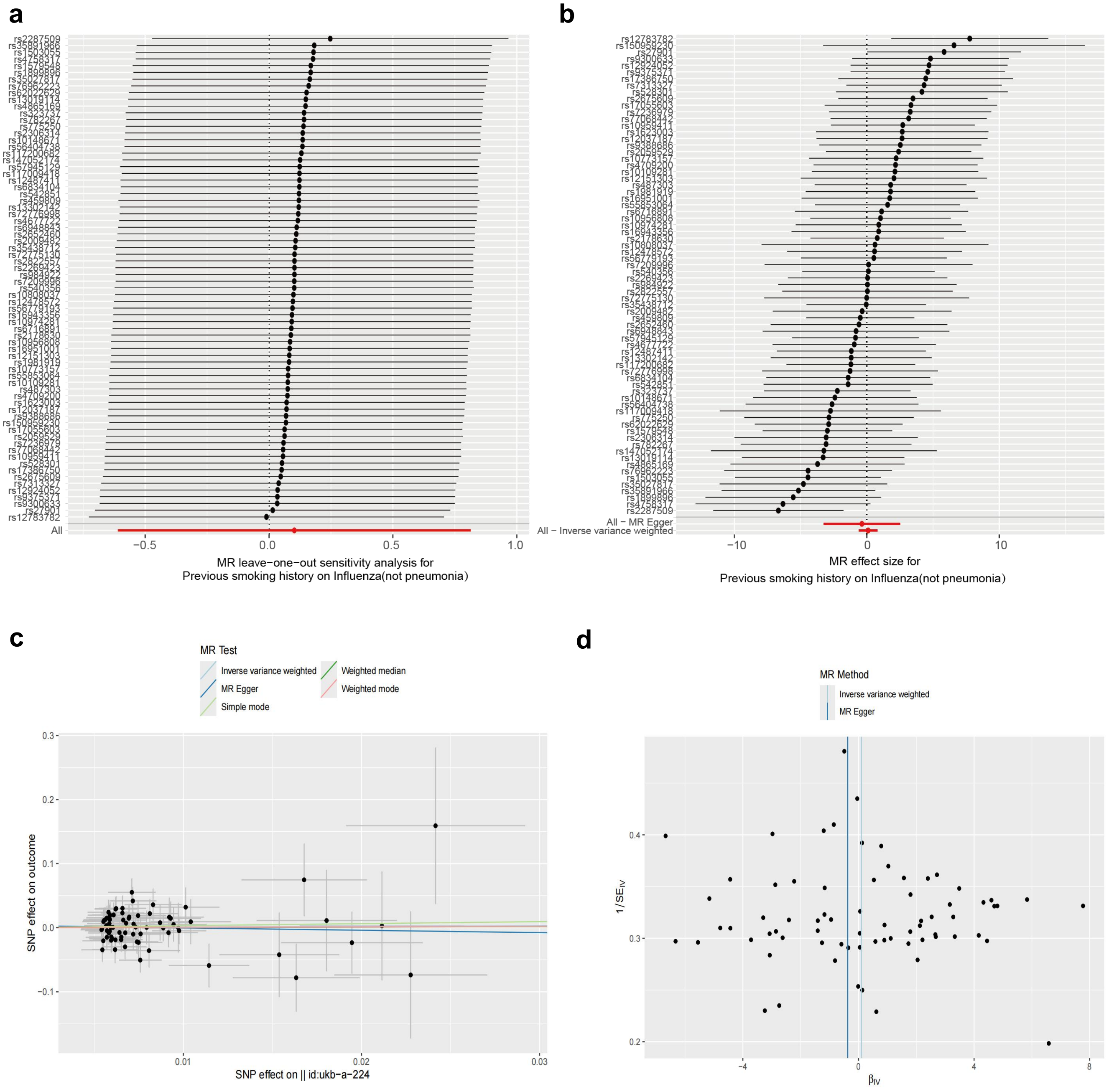

Figure S3.Mendelian randomization analysis of previous smoking history on the risk of influenza (excluding pneumonia). (a) Leave-one-out analysis of MR test from previous smoking history on influenza(not pneumonia). **(b)** Forest plot showing the effect estimates of individual SNPs associated with previous smoking history on the risk of influenza. **(c)** Regression lines representing MR test results for the causal effect of previous smoking history on influenza risk. **(d)**Funnel plot illustrating the distribution of individual SNP estimates for previous smoking history on influenza risk, used to assess potential bias or heterogeneity.
