# Supplementary material for: Causal Inference of Different Smoke Exposure Statuses and Influenza Risk: Insights From a Mendelian Randomization Study
Source: Clin Respir J. 2025 May 13;19(5):e70083. doi: 10.1111/crj.70083 (PMC12075745; doi:10.1111/crj.70083)
Supplement: Supplementary file 8 — Figure S4 Mendelian randomization analysis of previous smoking history on the risk of influenza and pneumonia. [file CRJ-19-e70083-s011.pdf]

Figure S4. Mendelian randomization analysis of previous smoking history on the risk of influenza and pneumonia.

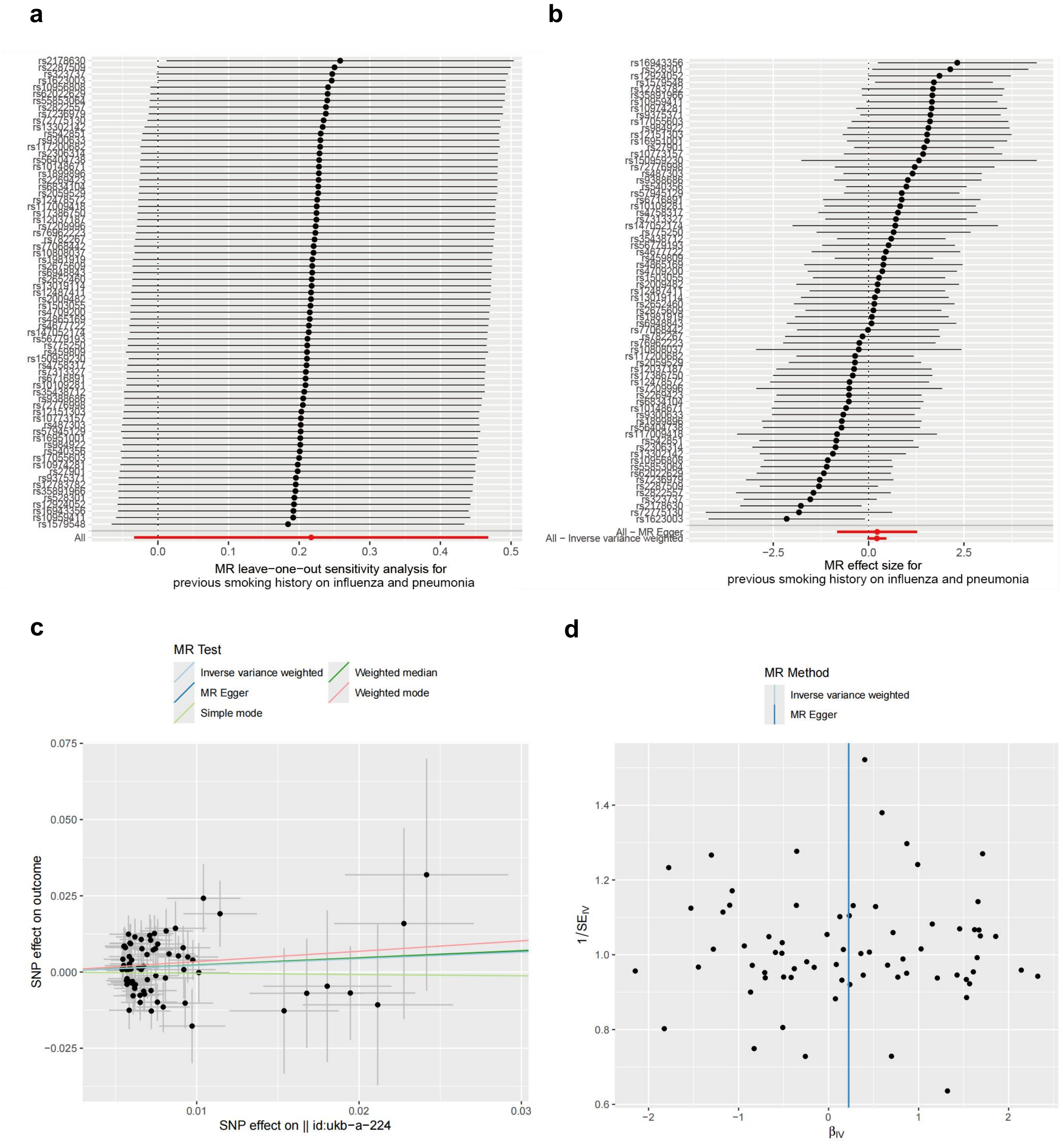

Figure S4.Mendelian randomization analysis of previous smoking history on the risk of influenza and pneumonia. (a) Leave-one-out analysis of MR test from previous smoking history on influenza and pneumonia. **(b)** Forest plot showing the effect estimates of individual SNPs associated with previous smoking history on the risk of influenza and pneumonia. **(c)** Regression lines representing MR test results for the causal effect of previous smoking history on influenza and pneumonia risk. **(d)** Funnel plot illustrating the distribution of individual SNP estimates for previous smoking history on influenza and pneumonia risk, used to assess potential bias or heterogeneity.
