# Supplementary material for: Causal Inference of Different Smoke Exposure Statuses and Influenza Risk: Insights From a Mendelian Randomization Study
Source: Clin Respir J. 2025 May 13;19(5):e70083. doi: 10.1111/crj.70083 (PMC12075745; doi:10.1111/crj.70083)
Supplement: Supplementary file 9 — Figure S5 Mendelian randomization analysis of household smoking exposure on the risk of influenza (excluding pneumonia). [file CRJ-19-e70083-s008.pdf]

Figure S5. Mendelian randomization analysis of household smoking exposure on the risk of influenza (excluding pneumonia).

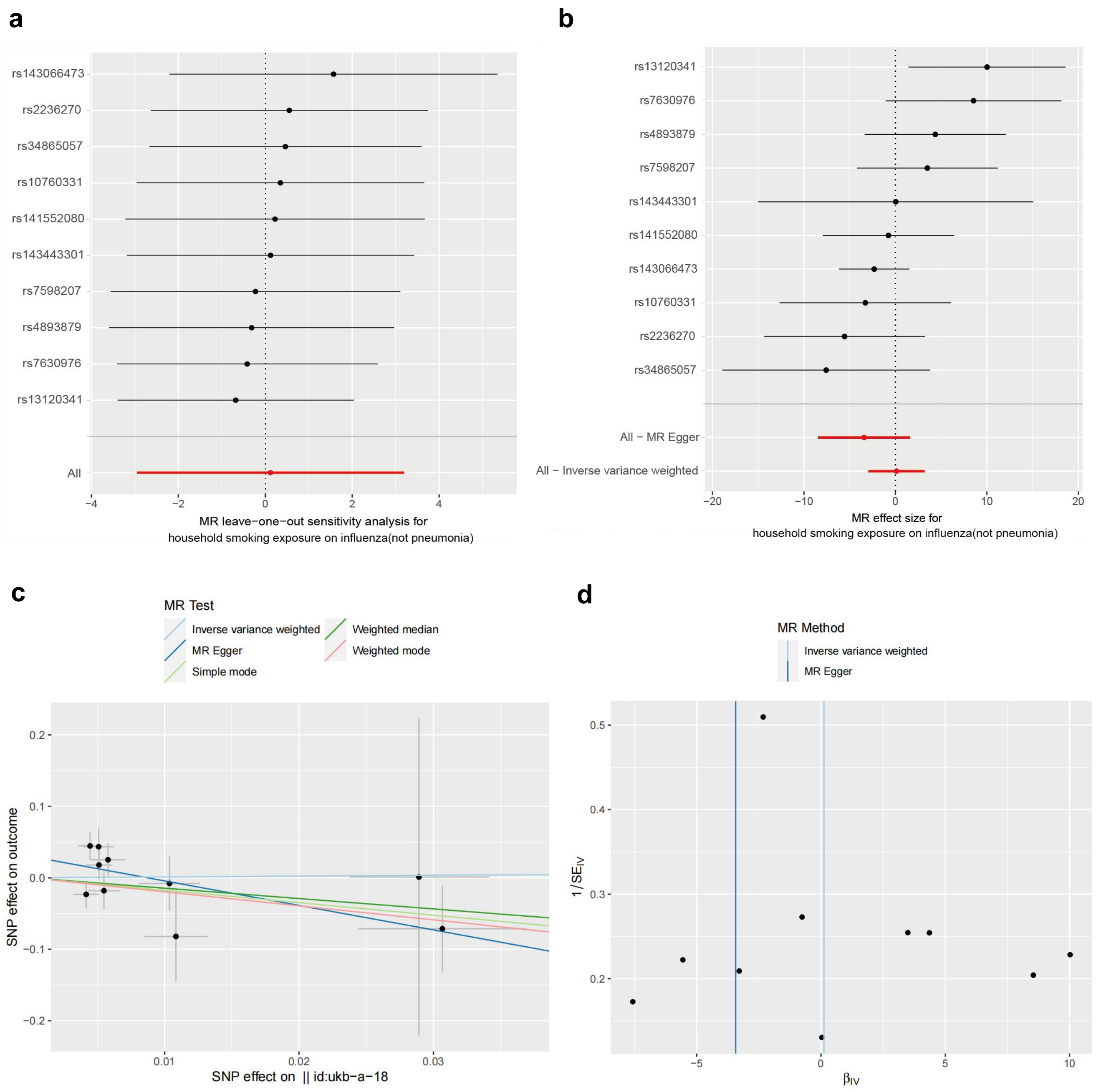

Figure S5. Mendelian randomization analysis of household smoking exposure on the risk of influenza (excluding pneumonia). (a) Leave-one-out analysis of MR test from household smoking exposure on influenza(not pneumonia). **(b)** Forest plot showing the effect estimates of individual SNPs associated with household smoking exposure on the risk of influenza. **(c)** Regression lines representing MR test results for the causal effect of household smoking exposure on influenza risk. **(d)** Funnel plot illustrating the distribution of individual SNP estimates for household smoking exposure on influenza risk, used to assess potential bias or heterogeneity.
