# Supplementary material for: Causal Inference of Different Smoke Exposure Statuses and Influenza Risk: Insights From a Mendelian Randomization Study
Source: Clin Respir J. 2025 May 13;19(5):e70083. doi: 10.1111/crj.70083 (PMC12075745; doi:10.1111/crj.70083)
Supplement: Supplementary file 10 — Figure S6 Mendelian randomization analysis of household smoking exposure on the risk of influenza and pneumonia. [file CRJ-19-e70083-s012.pdf]

Figure S6. Mendelian randomization analysis of household smoking exposure on the risk of influenza and pneumonia.

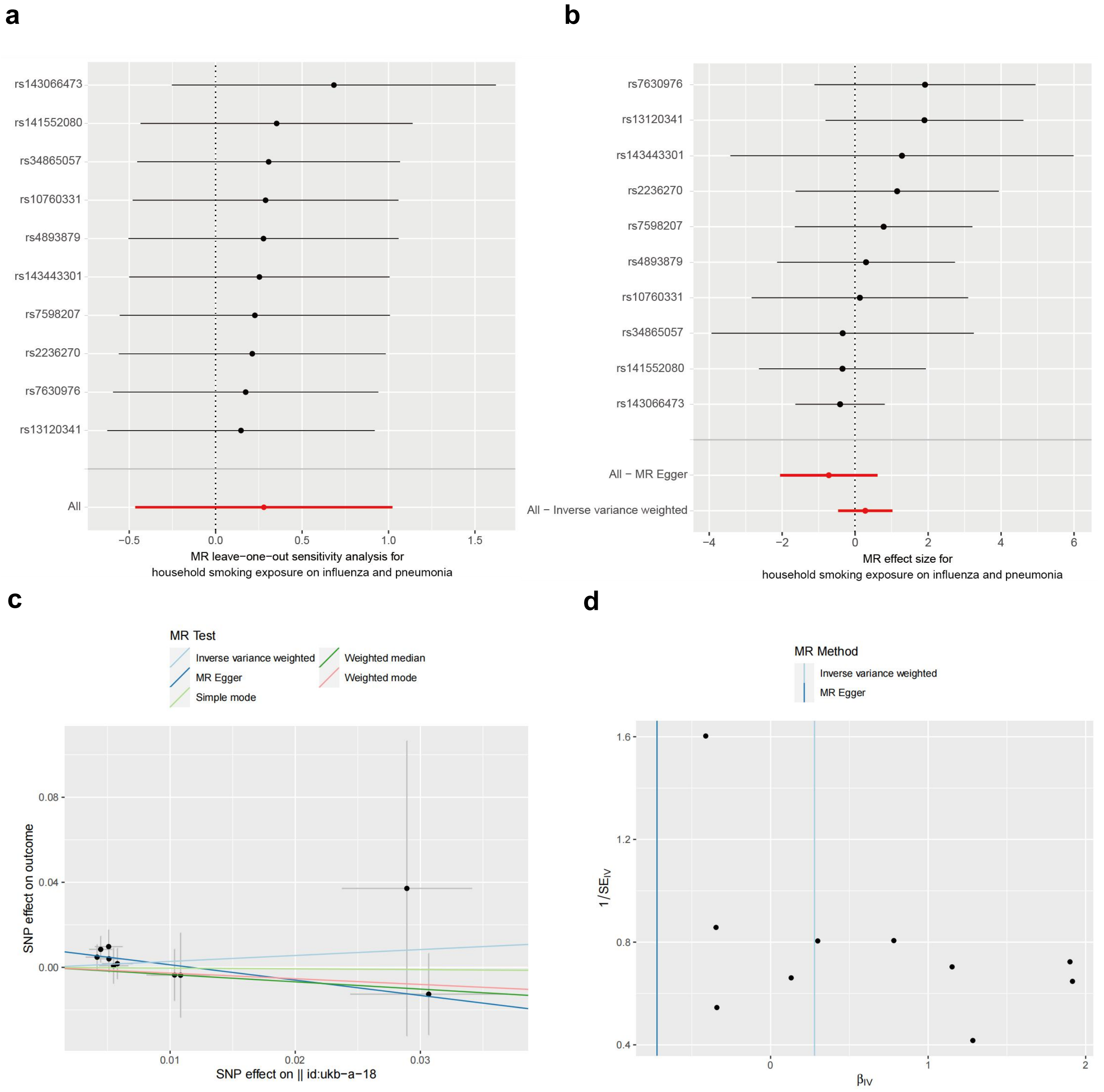

Figure S6. Mendelian randomization analysis of household smoking exposure on the risk of influenza and pneumonia.(a) Leave-one-out analysis of MR test from household smoking exposure on influenza and pneumonia. **(b)** Forest plot showing the effect estimates of individual SNPs associated with household smoking exposure on the risk of influenza and pneumonia. **(c)** Regression lines representing MR test results for the causal effect of household smoking exposure on influenza and pneumonia risk. **(d)** Funnel plot illustrating the distribution of individual SNP estimates for household smoking exposure on influenza and pneumonia risk, used to assess potential bias or heterogeneity.
