# Supplementary material for: Causal Inference of Different Smoke Exposure Statuses and Influenza Risk: Insights From a Mendelian Randomization Study
Source: Clin Respir J. 2025 May 13;19(5):e70083. doi: 10.1111/crj.70083 (PMC12075745; doi:10.1111/crj.70083)
Supplement: Supplementary file 11 — Figure S7 Mendelian randomization analysis of influenza (not pneumonia) infection on current tobacco use cohort. [file CRJ-19-e70083-s013.pdf]

Figure S7. Mendelian randomization analysis of influenza (not pneumonia) infection on current tobacco use cohort.

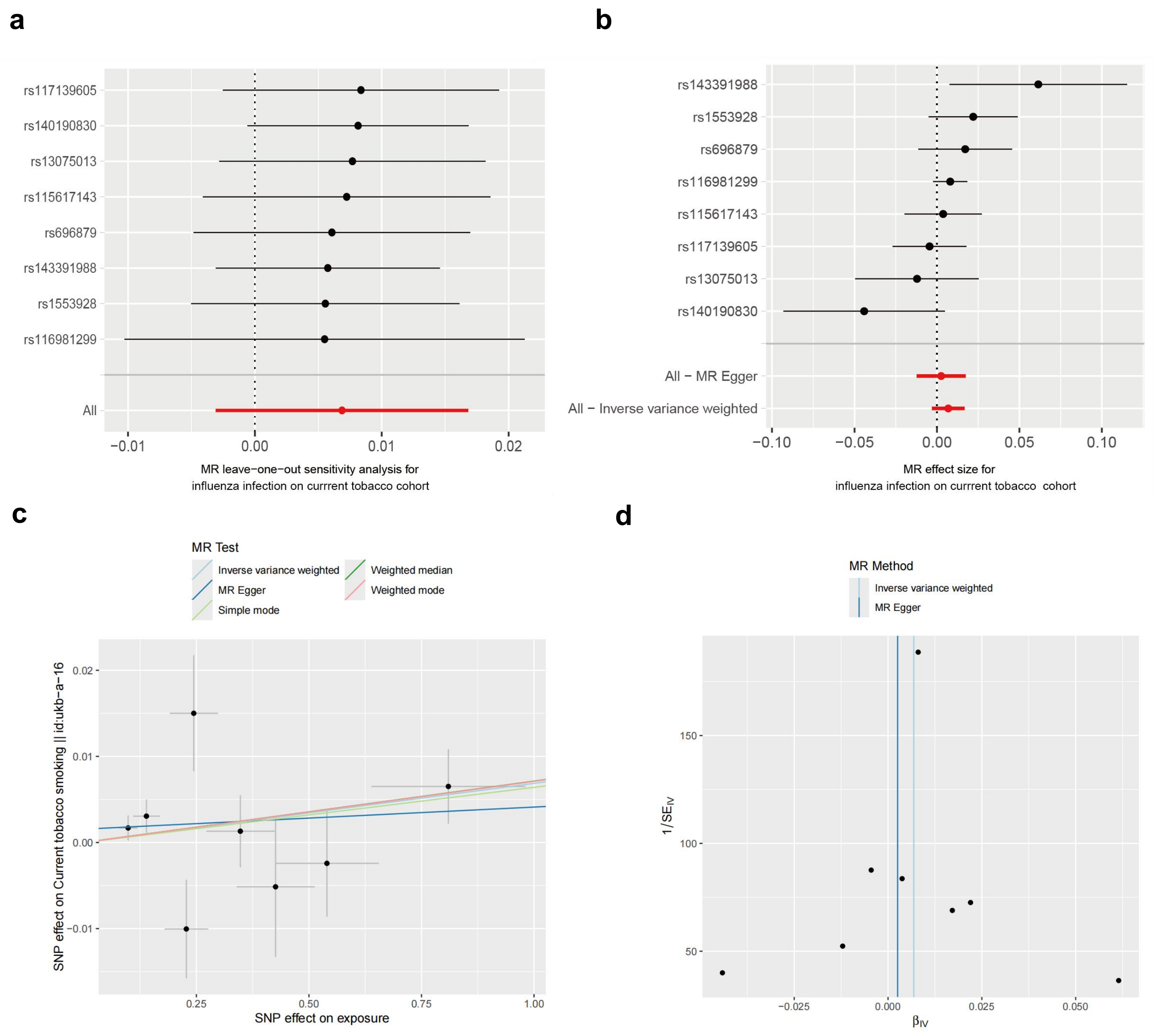

Figure S7. Mendelian randomization analysis of influenza (not pneumonia) infection on current tobacco use cohort. (a) Leave-one-out analysis of MR test from influenza infection on currrent tobacco cohort. **(b)**Forest plot showing the effect estimates of individual SNPs associated with influenza infection on currrent tobacco cohort. **(c)** Regression lines representing MR test results for the causal effect of influenza infection on current tobacco cohort. **(d)** Funnel plot illustrating the distribution of individual SNP estimates for influenza infection on currrent tobacco cohort, used to assess potential bias or heterogeneity.
