# Supplementary material for: Causal Inference of Different Smoke Exposure Statuses and Influenza Risk: Insights From a Mendelian Randomization Study
Source: Clin Respir J. 2025 May 13;19(5):e70083. doi: 10.1111/crj.70083 (PMC12075745; doi:10.1111/crj.70083)
Supplement: Supplementary file 15 — Figure S11 Mendelian randomization analysis of influenza and pneumonia infection on previous smoking cohort. [file CRJ-19-e70083-s007.pdf]

Figure S11. Mendelian randomization analysis of influenza and pneumonia infection on previous smoking cohort.

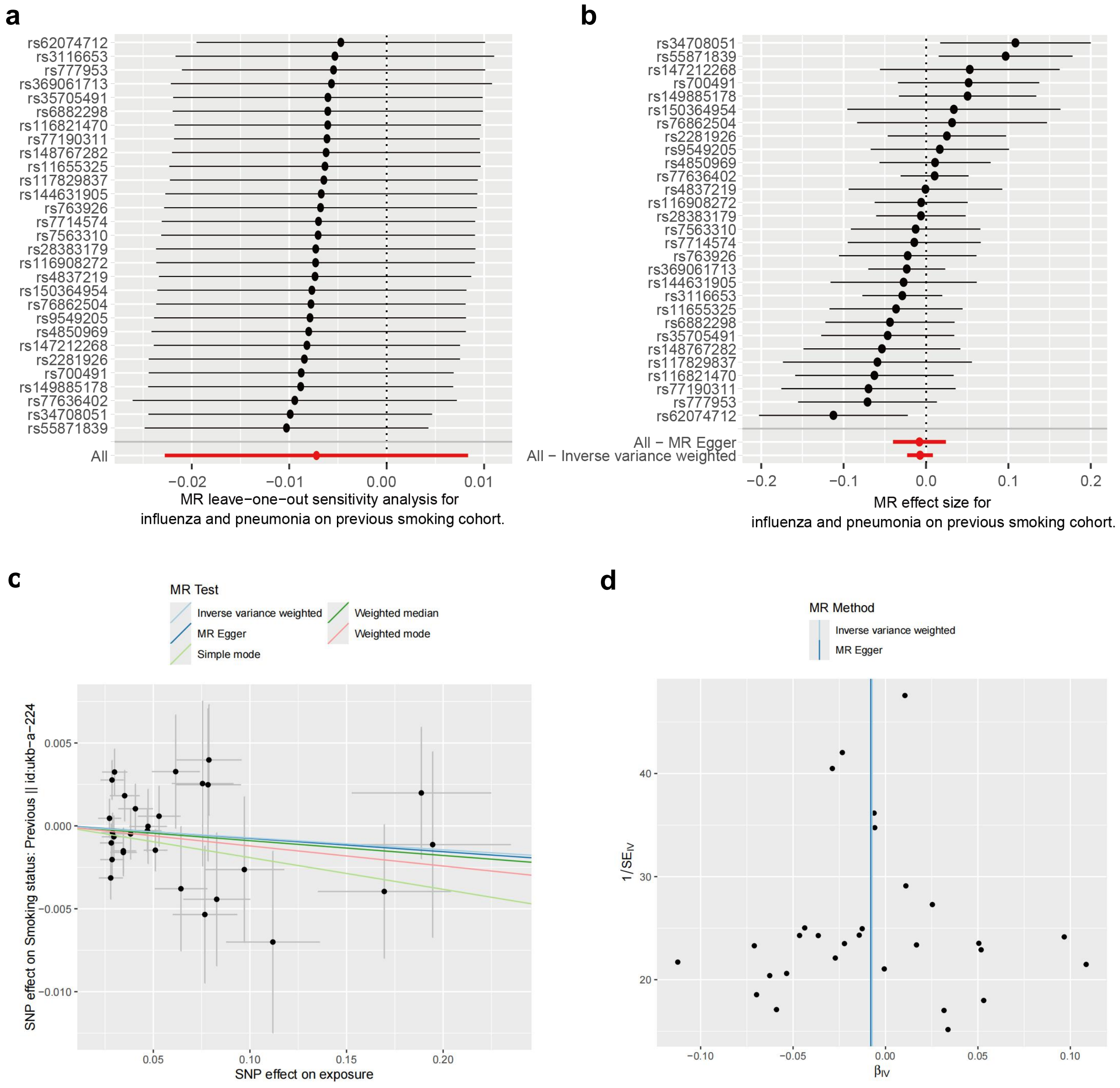

Figure S11. Mendelian randomization analysis of influenza and pneumonia infection on previous smoking cohort. (a) Leave-one-out analysis of MR test from influenza and pneumonia infection on previous smoking cohort. **(b)**Forest plot showing the effect estimates of individual SNPs associated with influenza and pneumonia infection on previous smoking cohort. **(c)** Regression lines representing MR test results for the causal effect of influenza and pneumonia infection on previous smoking cohort. **(d)** Funnel plot illustrating the distribution of individual SNP estimates for influenza and pneumonia infection on previous smoking cohort, used to assess potential bias or heterogeneity.
