# Supplementary material for: Causal Inference of Different Smoke Exposure Statuses and Influenza Risk: Insights From a Mendelian Randomization Study
Source: Clin Respir J. 2025 May 13;19(5):e70083. doi: 10.1111/crj.70083 (PMC12075745; doi:10.1111/crj.70083)
Supplement: Supplementary file 16 — Figure S12 Mendelian randomization analysis of influenza and pneumonia infection on household smoking exposure cohort. [file CRJ-19-e70083-s010.pdf]

Figure S12. Mendelian randomization analysis of influenza and pneumonia infection on household smoking exposure cohort.

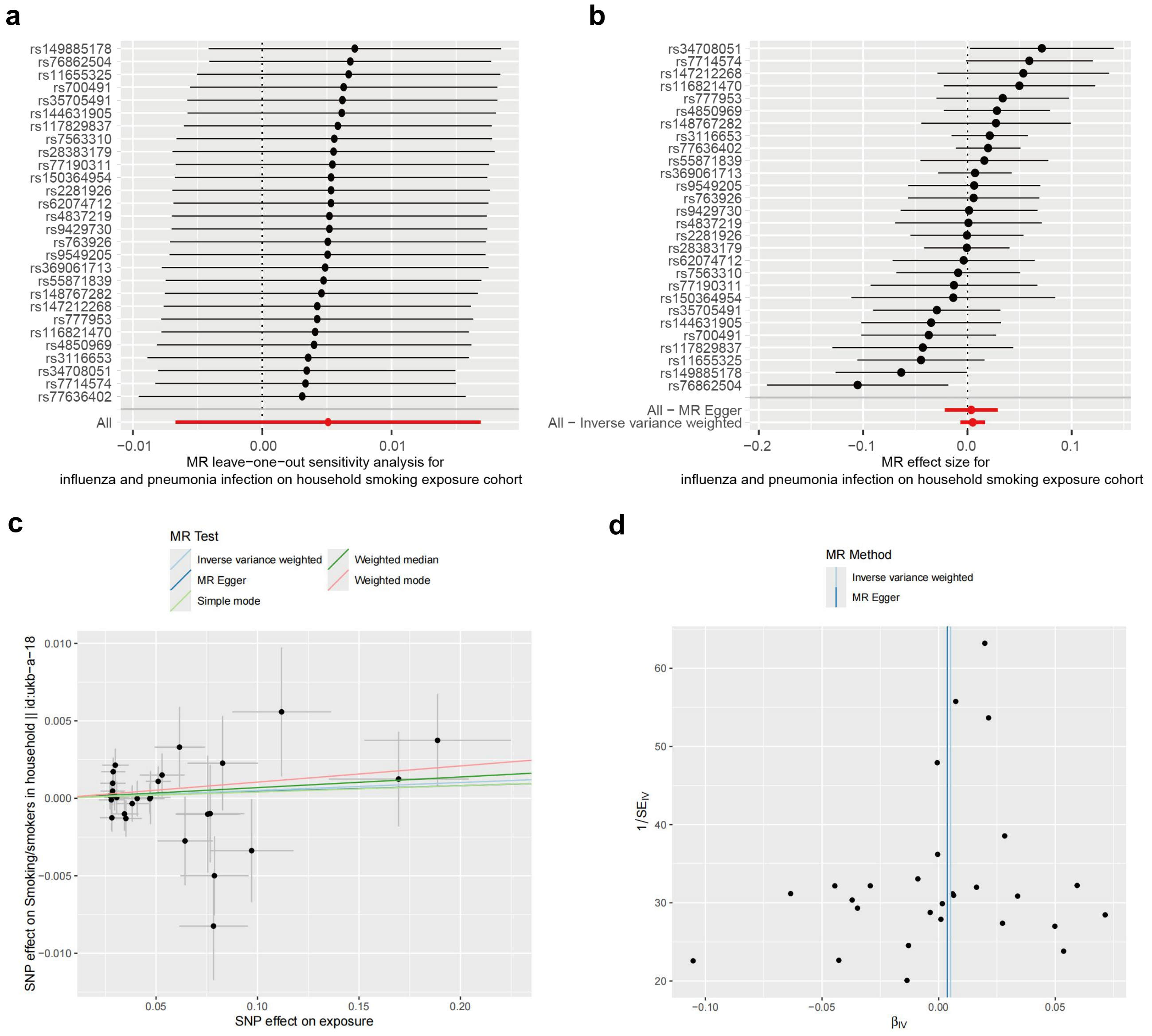

Figure S12. Mendelian randomization analysis of influenza and pneumonia infection on household smoking exposure cohort. (a) Leave-one-out analysis of MR test from influenza and pneumonia infection on household smoking exposure cohort. **(b)**Forest plot showing the effect estimates of individual SNPs associated with influenza and pneumonia infection on household smoking exposure cohort. **(c)** Regression lines representing MR test results for the causal effect of influenza and pneumonia infection on household smoking exposure cohort. **(d)** Funnel plot illustrating the distribution of individual SNP estimates for influenza and pneumonia infection on household smoking exposure cohort, used to assess potential bias or heterogeneity.
